# Comprehensive analysis of aberrantly expressed profiles of mRNA and its relationship with serum galactose-deficient IgA1 level in IgA nephropathy

**DOI:** 10.1186/s12967-019-2064-3

**Published:** 2019-09-23

**Authors:** Youxia Liu, Xiangchun Liu, Junya Jia, Jie Zheng, Tiekun Yan

**Affiliations:** 1 Department of Nephrology, Tianjin Medical University General Hospital, NO. 154, Anshan Road, Heping District, Tianjin, People’s Republic of China; 2 Department of Nephrology, The Second Hospital of Shandong University, Jinan, People’s Republic of China; 3 Radiology Department, Tianjin Medical University General Hospital, Tianjin, People’s Republic of China

**Keywords:** IgA nephropathy, mRNA microarray, Differential gene expression, RNA-deep sequencing, Galactose-deficient IgA1

## Abstract

**Background:**

Immunoglobulin A nephropathy (IgAN) is the leading cause of end-stage kidney disease. Previous mRNA microarray profiling studies of IgAN revealed inconsistent data. We sought to identify the aberrantly expressed genes and biological pathways by integrating IgAN gene expression datasets in blood cells and performing systematically experimental validation. We also explored the relationship between target genes and galactose-deficient IgA1 (Gd-IgA1) in IgAN.

**Methods:**

We retrieved Gene Expression Omnibus (GEO) datasets of IgAN. Gene Ontology (GO) enrichment and Kyoto Encyclopedia of Genes and Genomes (KEGG) analyses were used for functional analysis. Deep sequencing on RNA isolated from B cells was used for microarray validation. The relationship between target mRNA expressions and Gd-IgA1 levels in serum were also studied.

**Results:**

Three studies with microarray expression profiling datasets met our inclusion criteria. We identified 655 dyregulated genes, including 319 up-regulated and 336 down-regulated genes in three GEO datasets with a total of 35 patients of IgAN and 19 healthy controls. Based on biological process in GO term, these dyregulated genes are mainly related to pentose-phosphate shunt, non-oxidative branch, post-embryonic camera-type eye development and leukocyte activation. KEGG pathway analysis of microarray data revealed that these aberrantly expressed genes were enriched in human T-cell leukemia virus 1 infection, proteoglycans in cancer, intestinal immune network for IgA production and autophagy. We further performed deep sequencing on mRNAs isolated from B cells of an independent set of five patients with IgAN and three healthy persons with the same clinical and demographic characteristics. Seventy-seven genes overlapped with 655 differentially regulated genes mentioned above, including 43 up-regulated and thirty-four down-regulated genes. We next investigated whether these genes expression correlated with Gd-IgA1 levels in IgAN patients. Pearson correlation analyses showed *PTEN* (phosphatase and tensin homolog) was the most powerful gene negatively correlated with Gd-IgA1 levels.

**Conclusions:**

These results demonstrated that dyregulated genes in patients with IgAN were enriched in intestinal immune network for IgA production and autophagy process, and *PTEN* in B cells might be involved in the mechanism of Gd-IgA1 production.

## Background

Immunoglobulin A nephropathy (IgAN), also known as Berger’s disease, is the most common glomerulonephritis in the world [1], approximately 20% of such patients will progress to end-stage kidney disease (ESKD) within 10 years of diagnosis [2]. The initial event in the pathogenesis of IgAN is the production of galactose-deficient IgA1 (Gd-IgA1) [3], which lead to IgA1 self-aggregation, IgA1–IgG immune complex formation, and sequential deposition in the kidney [4, 5]. To date, the cause of IgAN remains to be determined.

Array-based gene-expression analysis is a useful tool to evaluate and identify differently expressed genes between pathological and control samples and is able to provide an unbiased screening and assessment of gene expression. Through microarray profiles, IgAN has been reported to be interrelated with multiple genes, including IFI27, *CXCL1* and *STAT3* [6–8]. However, recent studies showed that the data from one microarray analysis were inconsistent with other studies. The discrepancy may be due to small sample sizes and varying results obtained from different microarray platforms. Comprehensive gene set enrichment analysis could help us overcome these limitations and control such confounding factors by increasing statistical power, leading to more robust, reproducible and accurate predictions for identifying differently expressed genes [9, 10]. Indeed, such studies have been successfully used to identify gene signatures in many diseases [11–13].

In this study, we first identified differentially expressed genes and biological pathways in blood cells involved in IgAN using the chip data in Gene Expression Omnibus (GEO) database. Then deep sequencing on RNA isolated from B cells was used for microarray validation. We also explored the relationship between target genes and Gd-IgA1 in IgAN.

## Materials and methods

### Sample collection

A total of 25 IgAN patients diagnosed in Tianjin Medical University General Hospital from May to August 2017, were enrolled in this study. The diagnosis was based on the deposition of IgA in the glomerular mesangium by immunofluorescence detection, as well as the lack of clinical or serological evidence of other inflammatory conditions, such as Henoch Schoenlein purpura. At the same time, 21 healthy volunteers whose age and gender matched with patients were recruited. 5 IgAN patients and three healthy controls were included in the RNA deep sequencing experiment, 20 IgAN patients and 18 healthy controls were used for RNA deep sequencing validation. Written informed consent was obtained from each patient and healthy participant.

### Identification of individual studies and data processing

We performed an electronic search using the keywords ‘‘IgA nephropathy, IgA nephritis, or Berger’s disease’’ to identify studies that used microarray techniques through October 2018. We obtained mRNA expression data in GEO without language or publication date restrictions. Studies were included if they met the following criteria: (1) patients were diagnosed with IgAN based on IgAN diagnostic criteria [14]; (2) case–control studies; (3) all datasets were genome-wide; (4) all samples were from blood cells; (5) the number of cases and controls in each dataset must be ≥ 2; and (6) complete microarray raw data were available. We excluded any animal or duplicated studies. The whole genome raw expression data of included studies were downloaded from the National Center for Biotechnology Information GEO (http://www.ncbi.nlm.nih.gov/geo/). We used the Robust Multichip Average (RMA) algorithm in oligo package to normalize the raw expression data and generate normalized gene expression intensity [15]. After merging the data from different series, ComBat was used to adjust the known variances from different batches using an empirical Bayesian framework [16].

### Differential expression genes analysis

Differential expression gene analysis was performed using R v3.2.2. The tool based on a t-test was used to detect differentially expressed genes between IgAN patients and controls. The log2 transformation was used to obtain the standardized expression values [17]. Significantly up-regulated genes were defined by as a logarithmic transformed fold-change (FC) > 0.26 and P value ≤ 0.05. Significantly down-regulated genes were defined by a logFC ≤ 0.26 and P value ≤ 0.05.

### Functional analysis of differentially expressed genes

We next used Gene Ontology (GO) enrichment and Kyoto Encyclopedia of Genes and Genomes (KEGG) analyses to interpret biological significance of the differentially expressed genes. The gene-associated GO terms were used according to http://www.geneontology.org/ [18], while KEGG categories organized the differentially expressed genes into gene pathways (http://www.kegg.jp/) [19]. The functional analysis was performed using the using R v3.2.2.

### Assay for Gd-IgA1

Galactose-deficient IgA1 levels in serum were determined by ELISA. Serum Gd-IgA1 levels were detected using KM55 ELISA kit according to the manufacturer’s specifications (IBL, Japan) [20]. Serum samples were diluted in proportions of 1:50 with EIA buffer and incubation for 60 min with plate lid. Then washing four times with wash buffer, prepared labeled antibody was incubated for 30 min. Plates were washed and added 50 μL TMA solution incubation for 30 min in dark. At last, the color reaction was stopped and the absorbance was measured at 450/625 nm with an EL312 Bio-Kinetics microplate reader (Bio-Tek Instruments, Winooski, VT).

### B lymphocytes isolation

About 5 mL venous blood sample was taken into ethylenediaminetetraacetic acid (EDTA) anticoagulated tubes. Peripheral blood mononuclear cells (PBMCs) were separated by density-gradient centrifugation on Ficoll (TBD, China), then washed three times with phosphate buffered saline (PBS) and resuspended in PBS + 1% bovine serum albumin (BSA). Peripheral B lymphocytes were isolated using CD19 positive magnetic beads (Miltenyi Biotec, USA) according to the manufacturer’s instructions.

### RNA extraction and real time reverse transcription PCR (RT-PCR)

Total cellular RNA was extracted from CD19 positive B lymphocytes using the TRIZOL Reagent (Invitrogen, USA). RNA quantity was determined using NanoDrop ND-1000 spectrophotometer. cDNA was synthesized using 300 ng total RNA with revert first-strand cDNA kit according to manufacturer’s protocol (Promega, USA). Resulting cDNA was amplified with a 20 µL reaction mixture using SYBR Green PCR Master Mix (Roche, USA) in an Applied Biosystem 7500 Real-Time PCR System. And the primer pairs of *PTEN* and *GAPDH* were listed in Additional file 1: Table S1. The fold change between patients and controls was expressed by the 2^−△△CT^ method. The GAPDH gene amplification was used as a reference standard to normalize the target signal.

### RNA deep sequencing

RNA samples were sent to Shanghai Gminix, Biotechnology Co, Ltd. (Shanghai, China) for RNA deep sequencing on an Illumina HiSeq X10 sequencing platform with 15G PF data (Illumina, San Diego, CA). Procedures were performed as described in detail on the website of Gminix (http://www.gminix.com). The data discussed in this publication have been deposited in NCBI sequence read archive (SRA), and are accessible through SRA Series Accession number SUB6070993.

### Statistical analysis

The differences in gene expression levels between groups were compared using ANOVA and P value < 0.05 was considered statistically significant. Statistical analysis was performed using SPSS 17.0 software.

## Results

### Characteristics of included studies

**Fig. 1:**
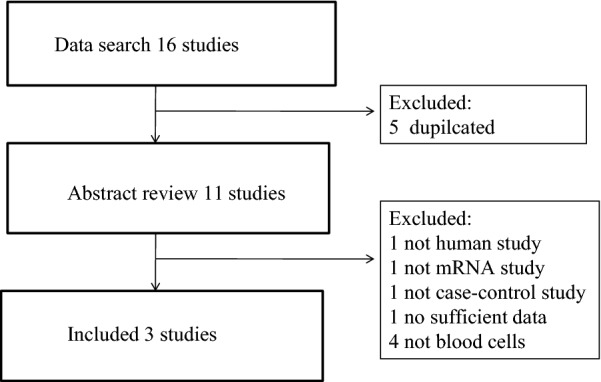
Flowchart for selection of eligible studies

We obtained 16 potential relevant publications and retrieved eleven studies for full text view following the initial database search. Three studies with microarray expression profiling data sets met our inclusion criteria (Fig. 1) [7, 21, 22]. The final analysis input consisted of 35 patients of IgAN and 19 healthy controls. Details of these selected studies are summarized in Table 1, including GEO accession, microarray platform, patients’ age, gender, and baseline levels of serum creatinine and proteinuria. These studies were published between 2010 and 2016 and sample size ranged from 17 to 20.

**Table 1:** Characteristics of the individual studies included in the meta-analysis

Study	Country	GEO accession	Platform	Sample	Numbers	Age	eGFR (mL/min/1.73 m^2^)	Proteinuria (g/days)
					IgAN/control			
Okuzaki, 2016	Japan	GSE73953	GPL4133	Blood cells	15/2	40/NA	NA	NA
Cox, 2015	Italy	GSE58539	GPL10558	Blood cells	8/9	40.1/33.4	115.3 ± 5.5/109.3 ± 4.3	0.33 ± 0.04/NA
Cox, 2010	Italy	GSE14795	GPL96	Blood cells	12/8	41.1/39.3	110.5 ± 10.7/106 ± 13.1	0.2 ± 0.02/0.1 ± 0.02

All data expressed as mean ± SD

### Differential gene expressions in IgAN patients

A total of 655 genes were identified differentially expressed in patients of IgAN group compared with controls across three microarray datasets (P < 0.05). Among these candidate implantation-associated genes, 319 were up-regulated and 336 were down-regulated in blood cells. A heat map visualization of the mRNA expression profile in IgAN was displayed in Additional file 2: Figure S1. We listed the top 10 most significantly up- or down-regulated genes in Table 2. The top 100 differentially expressed genes in GEO microarray were listed in Additional file 1: Table S2.

**Table 2:** The ten differentially expressed genes in IgAN patients and healthy control

Gene symbol	Gene name	P value	Fold_change
Downregulated			
GRPEL1	Proteasome subunit, beta type, 10	3.49 × 10^−5^	2.88
OAT	Poly (ADP-ribose) polymerase family, member 12	4.82 × 10^−5^	2.63
TFDP1	MMS19 nucleotide excision repair homolog (*S. cerevisiae*)	8.77 × 10^−5^	2.98
SLC25A24	Phosphopantothenoylcysteine decarboxylase	1.33 × 10^−4^	1.07
PPP1R2	Uncharacterized protein DKFZp586I1420	1.79 × 10^−4^	2.09
DDX21	Dynein, cytoplasmic 1, heavy chain 1	1.97 × 10^−4^	2.30
MBD2	Focadhesin	2.20 × 10^−4^	3.10
MAPRE1	Zinc finger protein 22	2.68 × 10^−4^	3.81
CXCR4	Exostosin-like glycosyltransferase 2	3.59 × 10^−4^	3.22
IL1A	KIAA0513	3.63 × 10^−4^	3.26
Upregulated			
DOK3	Docking protein 3	3.97 × 10^−6^	1.97
REEP4	Receptor accessory protein 4	9.99 × 10^−6^	2.21
MRPS18A	Mitochondrial ribosomal protein S18A	3.99 × 10^−5^	2.96
DEF8	Differentially expressed in FDCP 8 homolog	8.24 × 10^−5^	1.48
OXCT2	3-oxoacid CoA-transferase 2	2.19 × 10^−4^	1.74
MAN2B1	Mannosidase alpha class 2B member 1	3.26 × 10^−4^	3.46
LRP3	LDL receptor related protein 3	5.97 × 10^−4^	1.99
EDC4	Enhancer of mRNA decapping 4	7.97 × 10^−4^	2.17
RAB23	RAB23, member RAS oncogene family	8.48 × 10^−4^	1.88
COX4I1	Cytochrome *c* oxidase subunit 4I1	8.57 × 10^−4^	3.64

### Function analysis

**Fig. 2:**
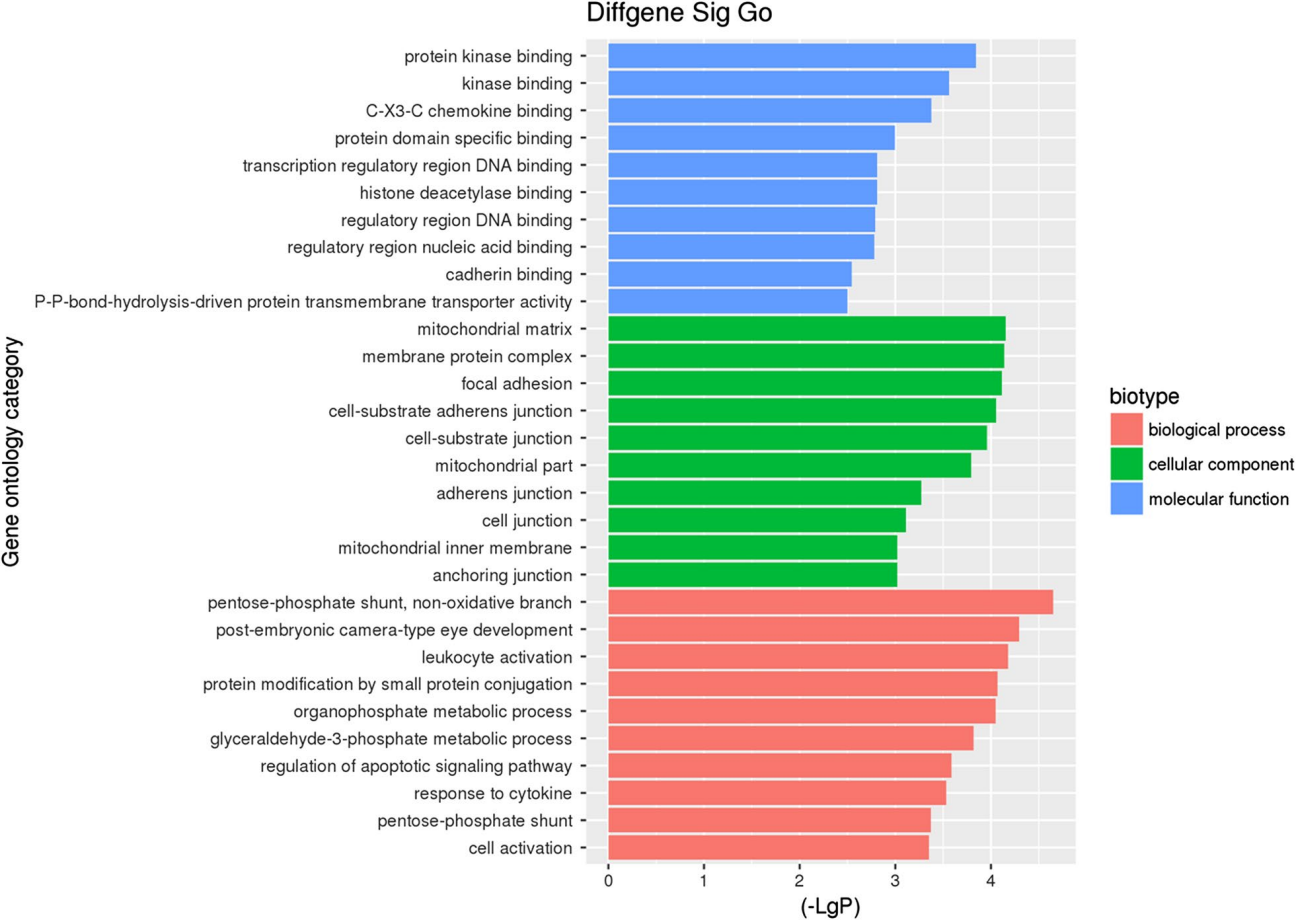
The top 10 most significantly enriched GO terms identified in IgAN

To analyze the related functions of the dyregulated genes, GO enrichment and KEGG pathway analysis were performed. After GO enrichment analysis, the top 10 most significant GO terms in biological process (BP), cellular component (CC), and molecular function (MF) were depicted in Fig. 2. Based on the BP, these dyregulated genes are mainly related to pentose-phosphate shunt, non-oxidative branch (GO: 0009052, four genes were enriched with P = 2.22 × 10^−5^), post-embryonic camera-type eye development (GO: 0031077, four genes were enriched with P = 5.05 × 10^−5^) and leukocyte activation (GO: 0045321, 69 genes were enriched with P = 6.60 × 10^−5^). For MF, the genes were mainly associated with protein kinase binding (GO: 0019901, 38 genes were enriched with P = 1.43 × 10^−4^), kinase binding (GO: 0019900, 41 genes were enriched with P = 2.75 × 10^−4^) and C-X3-C chemokine binding (GO: 0019960, three genes were enriched with P = 4.19 × 10^−4^). For CC, the genes were associated with mitochondrial matrix (GO: 0005759, 32 genes were enriched with P = 7.03 × 10^−5^), membrane protein complex (GO: 0098796, 42 genes were enriched with P = 7.29 × 10^−5^), and focal adhesion (GO: 0005925, 29 genes were enriched with P = 7.73 × 10^−5^).

**Fig. 3:**
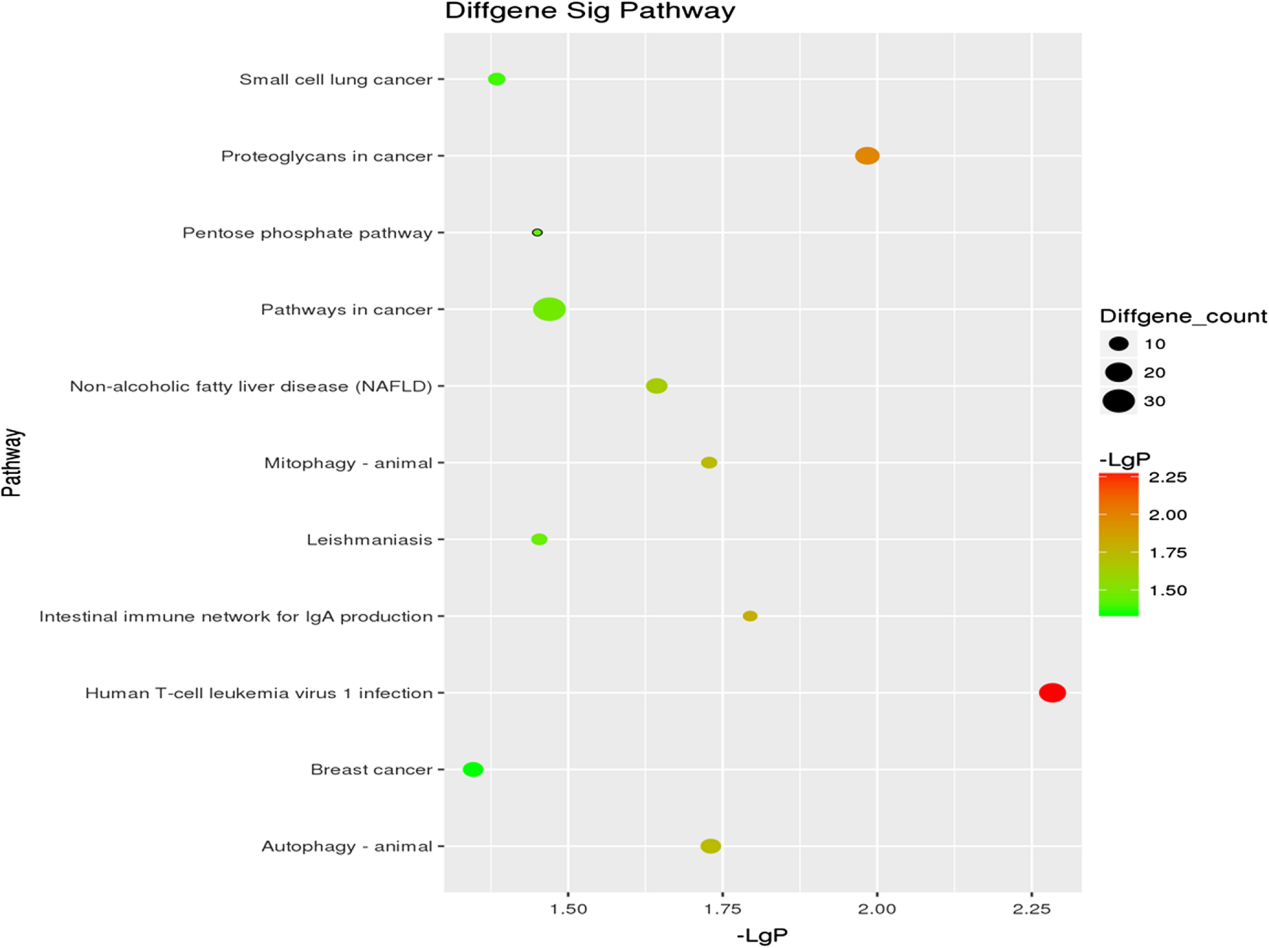
The top 10 most significant KEGG pathways identified in IgAN

Figure 3 shows the top 11 significantly enriched pathways in blood cells. The KEGG enrichment pathway analysis of our microarray data revealed that these dyregulated genes were enriched in human T-cell leukemia virus 1 infection (hsa05166, 20 genes were enriched with P = 0.005), proteoglycans in cancer (hsa05205, 16 genes were enriched with P = 0.01), intestinal immune network for IgA production (hsa04672, six genes were enriched with P = 0.01), autophagy-animal (hsa04140, 11 genes were enriched with P = 0.01) and mitophagy-animal (hsa04137, 7 genes were enriched with P = 0.01).

### Validation of selected targets

**Fig. 4:**
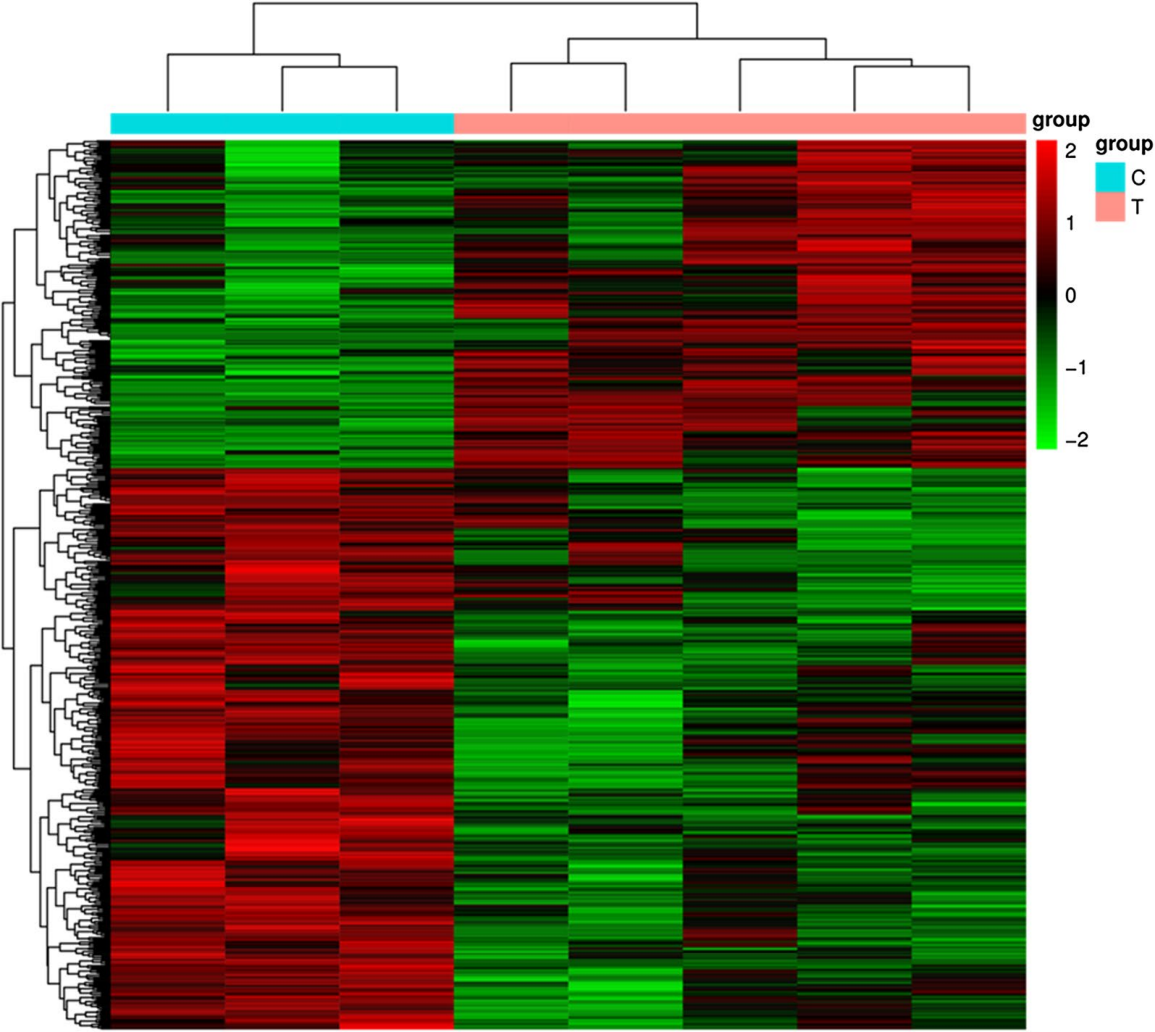
Heatmap illustration of the differentially expressed genes in IgAN. Expression levels are represented by red (high) and green (low expression). Samples are from five patients with IgAN (pink) and three healthy subjects (blue)

To further validate some of the results obtained, we performed deep sequencing on RNAs isolated from B cells of an independent set of five patients with IgAN and three healthy persons with the same clinical and demographic characteristics as those in the population used for microarray experiments (Table 3). A heat map visualization of the mRNA expression profile in IgAN was displayed in Fig. 4. The top 100 differential expression genes could be seen in Additional file 1: Table S3. We confirmed 77 differentially regulated genes, including 43 up-regulated and 34 down-regulated genes were overlapped with just identified 655 genes by microarray analysis. We listed 10 significantly up- or down-regulated genes in Table 4.

**Table 3:** Characteristics of individuals included for deep sequencing

Variable	IgAN	Healthy control
Participants (n)	5	3
Men/women (n/n)	3/2	2/1
Age (year)	39 ± 11	36 ± 13
Systolic BP (mmHg)	125 ± 10	121 ± 12
eGFR (mL/min/1.73 m^2^)	108.5 ± 9.8	NA
Proteinuria (g/24 h)	0.8 ± 0.3	NA

**Table 4:** Ten differentially expressed genes confirmed by deep sequencing

Gene symbol	Gene name	P for microarray	P for RNA-seq	Expression trend
MAN2B1	Mannosidase alpha class 2B member 1	3.26 × 10^−4^	0.04	Up
PTEN	Phosphatase and tensin homolog	5.95 × 10^−4^	0.03	Down
EDC4	Enhancer of mRNA decapping 4	7.98 × 10^−4^	0.01	Up
HES1	hes family bHLH transcription factor 1	8.08 × 10^−4^	0.02	Down
SLC39A6	Solute carrier family 39 member 6	1.48 × 10^−3^	0.01	Down
CD69	CD69 molecule	1.83 × 10^−3^	0.01	Down
B3GNTL1	UDP-GlcNAc:betaGal beta-1,3-*N*-acetylglucosaminyltransferase like 1	2.18 × 10^−3^	0.002	Up
PIAS3	Protein inhibitor of activated STAT 3	4.64 × 10^−3^	0.02	Up
ZNF41	Zinc finger protein 41	5.46 × 10^−3^	0.03	Down
CBFB	Core-binding factor subunit beta	6.06 × 10^−3^	0.04	Down

### Correlation between dyregulated genes expression and Gd-IgA1 levels

**Fig. 5:**
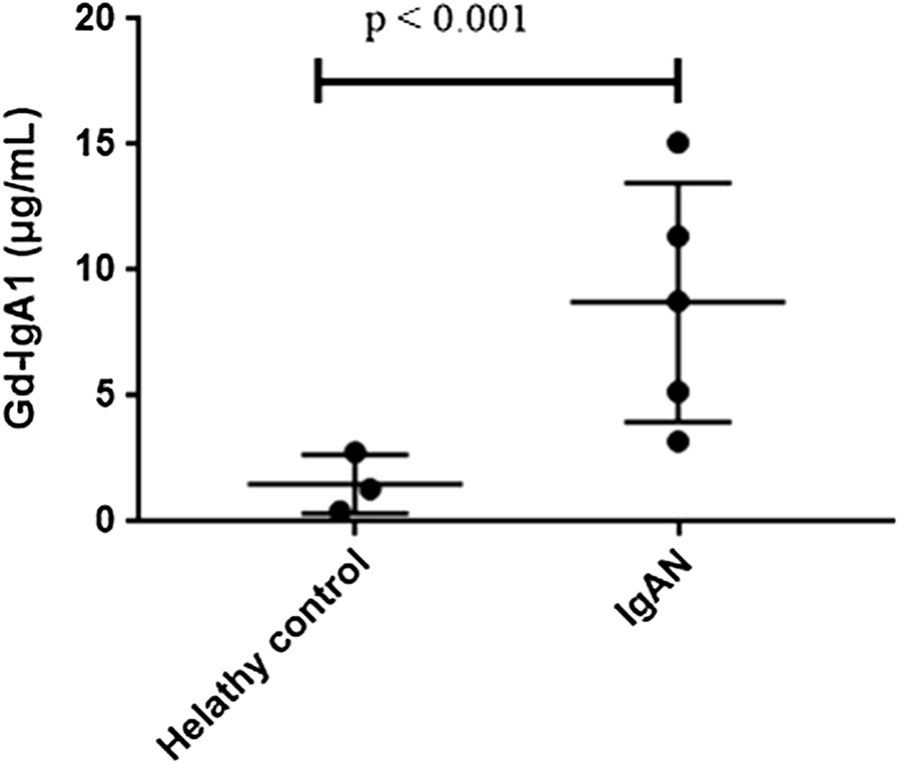
Serum Gd-IgA1 levels in IgAN patients and healthy control

**Fig. 6:**
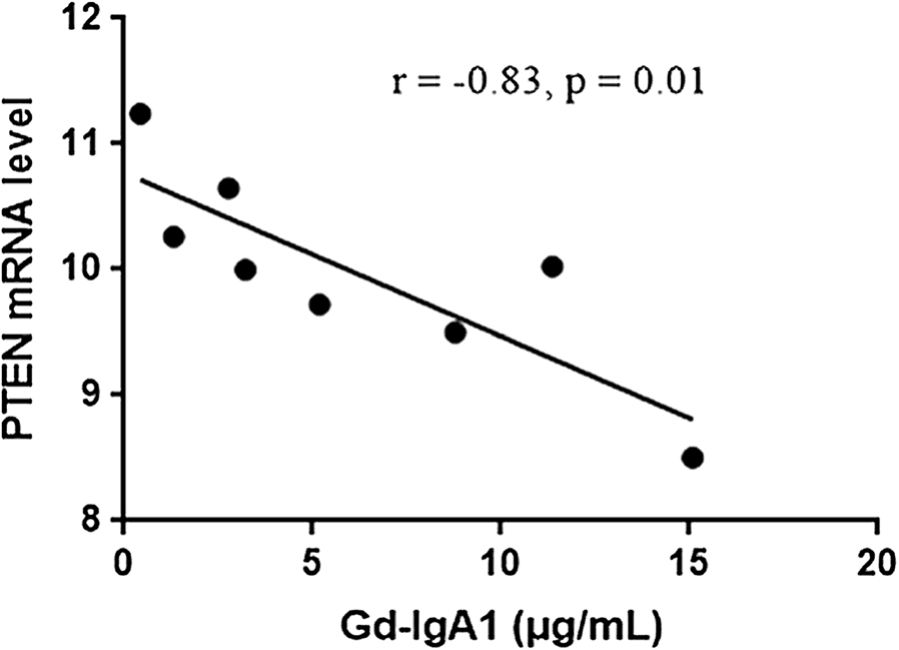
The correlation between PTEN and Gd-IgA1

After the confirmation that IgAN patients had abnormal expression of above 77 genes, we next investigated whether these genes expression was correlated with Gd-IgA1 levels in IgAN patients. Gd-IgA1 levels, evaluated by KM55 antibody, were significantly higher in serum of patients with IgAN than in those of healthy persons (P < 0.001; Fig. 5). The results identified eight genes whose expression was correlated with Gd-IgA1 levels (Additional file 1: Table 4). Pearson correlation analyses showed *PTEN* was the most powerful gene negatively correlated with Gd-IgA1 levels (r = − 0.83, P = 0.01, Fig. 6), suggesting that downregulated expression of PTEN might be involved in the mechanism of increased Gd-IgA1 production in IgAN.

### PTEN mRNA levels in IgAN patients

**Fig. 7:**
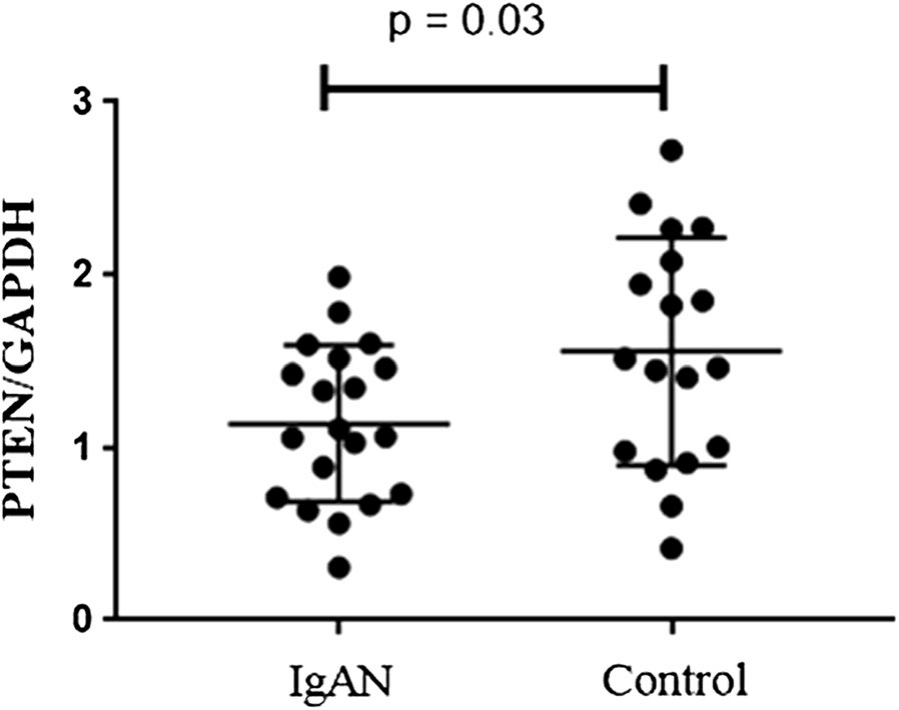
The PTEN mRNA level in 20 patients with IgAN and 18 healthy control

PTEN takes the 21th place in the top hits overall in microarray and 320th place in B-cell RNA-seq datasets. We next isolated CD19 + B lymphocytes from 20 IgAN patients to verify the differently expression of PTEN mRNA. As shown in Fig. 7, the PTEN mRNA level of patients with IgAN is significantly lower than that of healthy control (1.12 ± 0.47 vs. 1.54 ± 0.68, P = 0.03).

## Discussion

It is the first study that provides an overview of three gene expression arrays in blood cells in IgAN and eventually discovers genes and pathways potentially involved in IgAN pathogenesis. Deep sequencing on RNA further verified these dyregulated genes in B cells in IgAN patients compared to healthy control samples, supporting the validity of our results.

In the era of high-throughput techniques, integration of heterogeneous data of microarray holds the promise of providing new insights of pathogenesis of IgAN. Functional analysis helps to understand differentially expressed genes from the point of view of canonical prior knowledge structured in the form of pathways diagrams. Our study provided more insightful biological interpretation by performing GO enrichment and KEGG pathway analyses in IgAN patients and healthy persons. We found that human T-cell leukemia virus 1 infection, proteoglycans in cancer, intestinal immune network for IgA production and autophagy were involved in IgAN. Large international GWASs have identified several susceptibility loci involved in dynamic environment of the intestinal mucosa and abnormal mucosal production of IgA against microbial antigens associated with susceptibility to IgAN [23, 24]. When the significant loci were tested for enrichment in KEGG pathways, Krzysztof showed the top overrepresented pathway was “Intestinal Immune Network for IgA Production”, which played a central role in the disease pathogenesis [25]. These data are consistent with the clinical observation that intestinal mucosal infections frequently trigger episodes of IgAN [26]. The recent random controlled trial, the NEFIGAN study, was designed to evaluate the effectiveness of Nefecon, an oral formulation that releases glucocorticosteroid budesonide in the lower ileum and ascending colon [27]. The result showed that Nefecon significantly decreased the level of proteinuria and maintained stable kidney function in patients with IgAN. These results clearly linked intestinal mucosal inflammatory disorders with the risk of IgAN. Evidences suggested that the agents, including blisibimod and atacicept, which target increasing levels of circulating IgA, may provide more specific therapy than what is currently available.

Autophagy is a highly conserved process that degrades cellular long-lived proteins and organelles. Accumulating evidence indicates that autophagy plays a critical role in chronic kidney disease. Recent GWAS have suggested that *MTMR3* (encoding myotubularin-related phosphatase 3), participated in autophagy, is a susceptibility genes for IgAN [24]. And lower MTMR3 level was observed in IgAN patients in genome-wide gene expression analyses [28]. These data suggested autophagy may play an important role in the pathogenesis of IgAN.

Many genes tend to be expressed differentially in IgAN and the challenge is to identify the important genes and pathways associated with the disease. Characterizing the molecular and cellular events during the pathogenesis is an important endeavor. Recent studies indicated that Gd-IgA1 was the trigger factor of IgAN. After the confirmation 77 genes aberrantly expressed in IgAN by RNA sequence, we next investigated whether these genes expression was correlated with Gd-IgA1 levels in IgAN patients. The results identified eight genes whose expression was correlated with Gd-IgA1 levels. Pearson correlation analyses showed *PTEN* was the most powerful gene negatively correlated with Gd-IgA1 levels. *PTEN* (phosphatase and tensin homolog), has lipid/tyrosine phosphatase-dependent and -independent activities that alter cell behavior. Cox et al. [21] suggested that PTEN participated in the pathogenesis of IgAN. In line with our results, a previous study conducted on PBMCs of IgAN patients showed patients with IgAN exhibited lower PTEN expression [21, 29]. The precise function of this gene in production of Gd-IgA1 is still unclear. Grazia et al. demonstrated miR-148b significantly upregulated in patients with IgAN, and its putative target genes C1GALT1, INVS, and PTEN were down-regulated in these patients [30]. *C1GALT1*, encoding β1,3-galactosyltransferase, plays a critical role in the glycosylation process of IgA1 in IgAN patients [31, 32]. Whether there is a protein–protein interaction between C1GALT1 and PTEN or an abnormal upstream regulation mechanism is still unknown, future studies are needed to investigate the underlying molecular mechanisms of the gene alterations and develop novel strategies by targeting some of the identified genes/pathways as therapeutic tools to control IgAN.

Although many common susceptibility genes with IgAN were found by two methods, there were still several differential results between those previous microarray-analyses (discussed above) and our current RNA-seq analysis. Many top differentially expressed genes in IgAN patients and healthy control were not validated. The prevalence of IgAN shows differences among geographic and racial populations. The reported prevalence of IgAN is higher in Asia than in Europe and America. Association with the angiotensin-converting enzyme genes was observed in Asian but not in Western patients. Actually, most genetic association studies based on a candidate-gene approach have not been replicated between different races. Besides, genetic heterogeneity between ethnic groups, methodological problems (e.g., small sample size, different detection method) might be another reason contributing to this difference.

Despite being comprehensive, our analysis has several limitations. First, given that the extracted GEO datasets were based on comparisons of IgAN versus healthy controls, the predicted targets might not be specific to IgAN, but reflect general kidney disease. Second, the experiments took in whole-blood samples for gene-expression analyses were included, while those conducted in kidney biopsy samples were excluded. As we all know, Gd-IgA1 is mainly produced in B cells, which represents the first hit of the pathogenesis of IgAN. And in consideration of different transcriptase across tissues, we seek to identify the most robust different expressed genes in a blood cell transcriptome. Although we restricted input to blood cells datasets, some confounding factors (for example differences in blood cells types, chip platform, cohort sizes or patients’ ethnicity) are not taken into account, which collectively may affect the analysis output. Third, it is unclear how far the results of our analysis may be generalized to the world population of patients with IgAN, there were only eight Chinese subjects included for experimental validation. Further studies with larger sample sizes are needed to identify target mRNA and assess its relationship with serum galactose-deficient IgA1 level in IgAN.

## Conclusion

In conclusion, these results demonstrated that dyregulated genes in blood cells of patients with IgAN are enriched in intestinal immune network for IgA production and autophagy. *PTEN* might be involved in the mechanism of increased Gd-IgA1 production in IgAN. Such altered genes and pathways could helpfully expand our knowledge of IgAN pathogenesis.

## Supplementary information

**Additional file 1: Table S1.** Primers used to amplify the *PTEN* and *GAPDH* genes. **Table S2.** The top 100 differentially expressed genes in GEO microarray. **Table S3.** The top 100 differentially expressed genes by RNA-deep sequencing. **Table S4.** Correlation between 8 dyregulated genes expression and Gd-IgA1 levels.

**Additional file 2: Figure S1.** Heatmap illustration of the patterns of change in a particular gene across different datasets. Expression levels are represented by red (high) and green (low expression). Samples are from 35 patients with IgAN (red) and 19 healthy subjects (green).

## Data Availability

Raw data used during the current study are available from the corresponding author on reasonable request for non-commercial use.

## Abbreviations

IgAN: immunoglobulin A nephropathy
GEO: Gene Expression Omnibus
GO: Gene Ontology
KEGG: Kyoto Encyclopedia of Genes and Genomes
Gd-IgA1: galactose-deficient IgA1
ESKD: end-stage kidney disease
PTEN: phosphatase and tensin homolog
