# Supplementary material for: Comprehensive analysis of aberrantly expressed profiles of mRNA and its relationship with serum galactose-deficient IgA1 level in IgA nephropathy
Source: J Transl Med. 2019 Sep 23;17:320. doi: 10.1186/s12967-019-2064-3 (PMC6757375; doi:10.1186/s12967-019-2064-3)
Supplement: Supplementary file 1 — Additional file 1: Table S1. Primers used to amplify the PTEN and GAPDH genes. Table S2. The top 100 differentially expressed genes in GEO microarray. Table S3. The top 100 differentially expressed genes by RNA-deep sequencing. Table S4. Correlation between 8 dyregulated genes expression and Gd-IgA1 levels. [file 12967_2019_2064_MOESM1_ESM.docx]

**Table S1. Primers used to amplify the *PTEN* and *GAPDH* genes.**

| Gene symbol | Forward Primers (5‘-3’) | Reverse Primers (5‘-3’) |
| --- | --- | --- |
| *PTEN* | TTTGAAGACCATAACCCACCAC | ATTACACCAGTTCGTCCCTTTC |
| *GAPDH* | TTGCCCTCAACGACCACTTT | TGGTCCAGGGGTCTTACTCC |

**Table S2. The top 100 differentially expressed genes in GEO microarray.**

| Gene Symbol | P |
| --- | --- |
| DOK3 | 3.98E-06 |
| REEP4 | 9.99E-06 |
| GRPEL1 | 3.49E-05 |
| MRPS18A | 3.99E-05 |
| OAT | 4.82E-05 |
| DEF8 | 8.24E-05 |
| TFDP1 | 8.77E-05 |
| SLC25A24 | 0.000133193 |
| PPP1R2 | 0.000179038 |
| DDX21 | 0.000196528 |
| OXCT2 | 0.00021961 |
| MBD2 | 0.000219734 |
| MAPRE1 | 0.00026785 |
| MAN2B1 | 0.000326864 |
| CXCR4 | 0.000358668 |
| IL1A | 0.000362771 |
| CYTIP | 0.000408807 |
| COQ10B | 0.000418433 |
| MEX3C | 0.000558429 |
| DOCK9 | 0.000572013 |
| PTEN | 0.000595111 |
| LRP3 | 0.0005972 |
| ARID5B | 0.000774199 |
| EDC4 | 0.000797999 |
| HES1 | 0.000808934 |
| MARCKSL1 | 0.000809646 |
| ADAM10 | 0.000821228 |
| RCN2 | 0.000847327 |
| RAB23 | 0.000848907 |
| COX4I1 | 0.000857766 |
| TOMM20 | 0.000903711 |
| FCGRT | 0.0009067 |
| EPHB6 | 0.00093344 |
| YWHAQ | 0.000940242 |
| TCEA1 | 0.000961918 |
| KRI1 | 0.000980379 |
| TMEM123 | 0.001034523 |
| MOAP1 | 0.001040516 |
| PTGER2 | 0.001053632 |
| OGFR | 0.001151438 |
| MSRB2 | 0.001173271 |
| INPPL1 | 0.001209126 |
| FBXL15 | 0.001225996 |
| PIGP | 0.001285409 |
| CHMP2B | 0.001346351 |
| RBBP6 | 0.001413525 |
| NPTN | 0.001467155 |
| PIK3R2 | 0.001469015 |
| SLC39A6 | 0.00148195 |
| FADD | 0.001647297 |
| TSPYL1 | 0.001704835 |
| LYRM2 | 0.00175532 |
| OXSR1 | 0.00177841 |
| CD69 | 0.001838887 |
| DHRS7B | 0.001842412 |
| TMEM144 | 0.001935828 |
| UBE2E3 | 0.001968307 |
| BLZF1 | 0.002012549 |
| NAGK | 0.002043185 |
| MGAT1 | 0.002119673 |
| ACTR1A | 0.002141951 |
| TMEM11 | 0.002143277 |
| PRPF38B | 0.002164196 |
| ARL6IP4 | 0.002170335 |
| LMAN1 | 0.002184134 |
| B3GNTL1 | 0.002188029 |
| CDKN1A | 0.002232287 |
| DDX28 | 0.002255874 |
| SMPD3 | 0.002283055 |
| GCH1 | 0.002389432 |
| IP6K1 | 0.00242899 |
| THUMPD1 | 0.0024359 |
| MORF4L1 | 0.00249835 |
| ETV6 | 0.002565379 |
| PNRC2 | 0.002633732 |
| TST | 0.002634834 |
| NFE2 | 0.002674942 |
| ACYP2 | 0.002688951 |
| ARF4 | 0.002711457 |
| XPNPEP1 | 0.002733502 |
| HSPA6 | 0.002784451 |
| CSF2RB | 0.002843191 |
| C2orf47 | 0.002853984 |
| EPS15L1 | 0.002867393 |
| ENPP4 | 0.002897702 |
| OPN3 | 0.002951291 |
| HSD17B11 | 0.002999511 |
| ARHGAP26 | 0.003021144 |
| COG4 | 0.003022894 |
| EIF1B | 0.003061547 |
| HPRT1 | 0.003101275 |
| RPS10 | 0.003110146 |
| PDZD8 | 0.003110162 |
| ALMS1 | 0.003183409 |
| THAP10 | 0.00319587 |
| IARS | 0.003218973 |
| HIST2H2BE | 0.003245387 |
| AZIN1 | 0.00337003 |
| OXSM | 0.003380304 |
| APBB3 | 0.003392631 |

| **Table S3. The top 100 differentially expressed genes by** RNA-**deep**  **sequencing.** |
| --- |
| \| Gene Symbol \| P \| \| --- \| --- \| \| PEX11B \| 3.77E-07 \| \| HMG20B \| 1.25E-06 \| \| PLCXD2 \| 6.64E-06 \| \| GPR61 \| 6.91E-06 \| \| SLC13A4 \| 6.93E-06 \| \| FAM78A \| 1.27E-05 \| \| CNDP2 \| 1.37E-05 \| \| NRSN2 \| 1.40E-05 \| \| NPHP4 \| 1.73E-05 \| \| TTYH3 \| 4.43E-05 \| \| DHDDS \| 8.00E-05 \| \| TNRC18 \| 8.49E-05 \| \| SULF2 \| 0.000113 \| \| MAPK3 \| 0.000113 \| \| C2orf81 \| 0.000121 \| \| MAP3K3 \| 0.000149 \| \| OLA1 \| 0.000156 \| \| CCDC90B \| 0.000187 \| \| AP4M1 \| 0.000198 \| \| PLCXD2 \| 0.000225 \| \| TBL1XR1 \| 0.000232 \| \| CHSY1 \| 0.000237 \| \| VASH2 \| 0.00027 \| \| PTPN11 \| 0.000291 \| \| DOK7 \| 0.000308 \| \| RAB22A \| 0.000336 \| \| MIA3 \| 0.000363 \| \| MGAT1 \| 0.000364 \| \| GPSM3 \| 0.00038 \| \| CHORDC1 \| 0.000453 \| \| PARP12 \| 0.000475 \| \| CRYZL1 \| 0.00048 \| \| HDHD2 \| 0.000482 \| \| ARHGAP24 \| 0.000482 \| \| PCBP2 \| 0.000482 \| \| ZEB1 \| 0.000496 \| \| EMG1 \| 0.000502 \| \| BRI3BP \| 0.000511 \| \| UBXN1 \| 0.000525 \| \| KIAA1143 \| 0.000554 \| \| EIF3B \| 0.000555 \| \| ARPC2 \| 0.000562 \| \| NDEL1 \| 0.000621 \| \| PSMD7 \| 0.000636 \| \| DDA1 \| 0.00066 \| \| MCC \| 0.000687 \| \| U2AF2 \| 0.000699 \| \| TBL1XR1 \| 0.0007 \| \| SHPRH \| 0.000729 \| \| PAAF1 \| 0.000757 \| \| STK33 \| 0.000766 \| \| IL6ST \| 0.000769 \| \| LGR6 \| 0.000778 \| \| NUP214 \| 0.000789 \| \| WDR76 \| 0.000794 \| \| RABL2B \| 0.000817 \| \| TADA2A \| 0.000839 \| \| RBM6 \| 0.00085 \| \| BRE \| 0.000854 \| \| C6orf163 \| 0.000857 \| \| ZNF217 \| 0.000894 \| \| C1orf109 \| 0.000905 \| \| CNOT3 \| 0.000928 \| \| RPP38 \| 0.000932 \| \| ZNF548 \| 0.000936 \| \| AGBL5 \| 0.000948 \| \| C8G \| 0.001002 \| \| COTL1 \| 0.001005 \| \| SUPT20H \| 0.001011 \| \| UNC93B1 \| 0.001021 \| \| PROSER3 \| 0.00105 \| \| CD44 \| 0.001053 \| \| U2AF2 \| 0.001072 \| \| MED6 \| 0.001094 \| \| HDHD2 \| 0.001108 \| \| RPS15A \| 0.001158 \| \| ZNF724P \| 0.001167 \| \| SERPINB8 \| 0.001177 \| \| TRAPPC11 \| 0.001237 \| \| IVNS1ABP \| 0.00124 \| \| CBX3 \| 0.00125 \| \| PLEKHA4 \| 0.001255 \| \| SSSCA1 \| 0.001323 \| \| RSBN1 \| 0.001346 \| \| CAPN1 \| 0.001357 \| \| TTN \| 0.001371 \| \| USF1 \| 0.001412 \| \| NSMCE2 \| 0.001478 \| \| PDE4DIP \| 0.00149 \| \| CEP85 \| 0.001508 \| \| RHBDF2 \| 0.001521 \| \| ORC3 \| 0.00153 \| \| ATXN7L1 \| 0.001533 \| \| TIAM2 \| 0.001606 \| \| JKAMP \| 0.001664 \| \| DDX59 \| 0.001672 \| \| DUSP5 \| 0.001674 \| \| TNIP1 \| 0.001689 \| \| OSM \| 0.001704 \| \| UBE3A \| 0.001708 \| |

**Table S4. Correlation between 8 dyregulated genes expression and Gd-IgA1 levels.**

| Gene symbol | r | P |
| --- | --- | --- |
| CD69 | 0.50 | 0.03 |
| KLF10 | 0.42 | 0.04 |
| SLC39A6 | 0.39 | 0.02 |
| PTEN | -0.83 | 0.01 |
| DDRGK1 | -0.65 | 0.01 |
| B3GNTL1 | -0.51 | 0.02 |
| CBFB | -0.41 | 0.04 |
| EMG1 | -0.40 | 0.04 |
