# Supplementary figures and images for: Comprehensive analysis of aberrantly expressed profiles of mRNA and its relationship with serum galactose-deficient IgA1 level in IgA nephropathy
Source: J Transl Med. 2019 Sep 23;17:320. doi: 10.1186/s12967-019-2064-3 (PMC6757375; doi:10.1186/s12967-019-2064-3)

## Slide 1
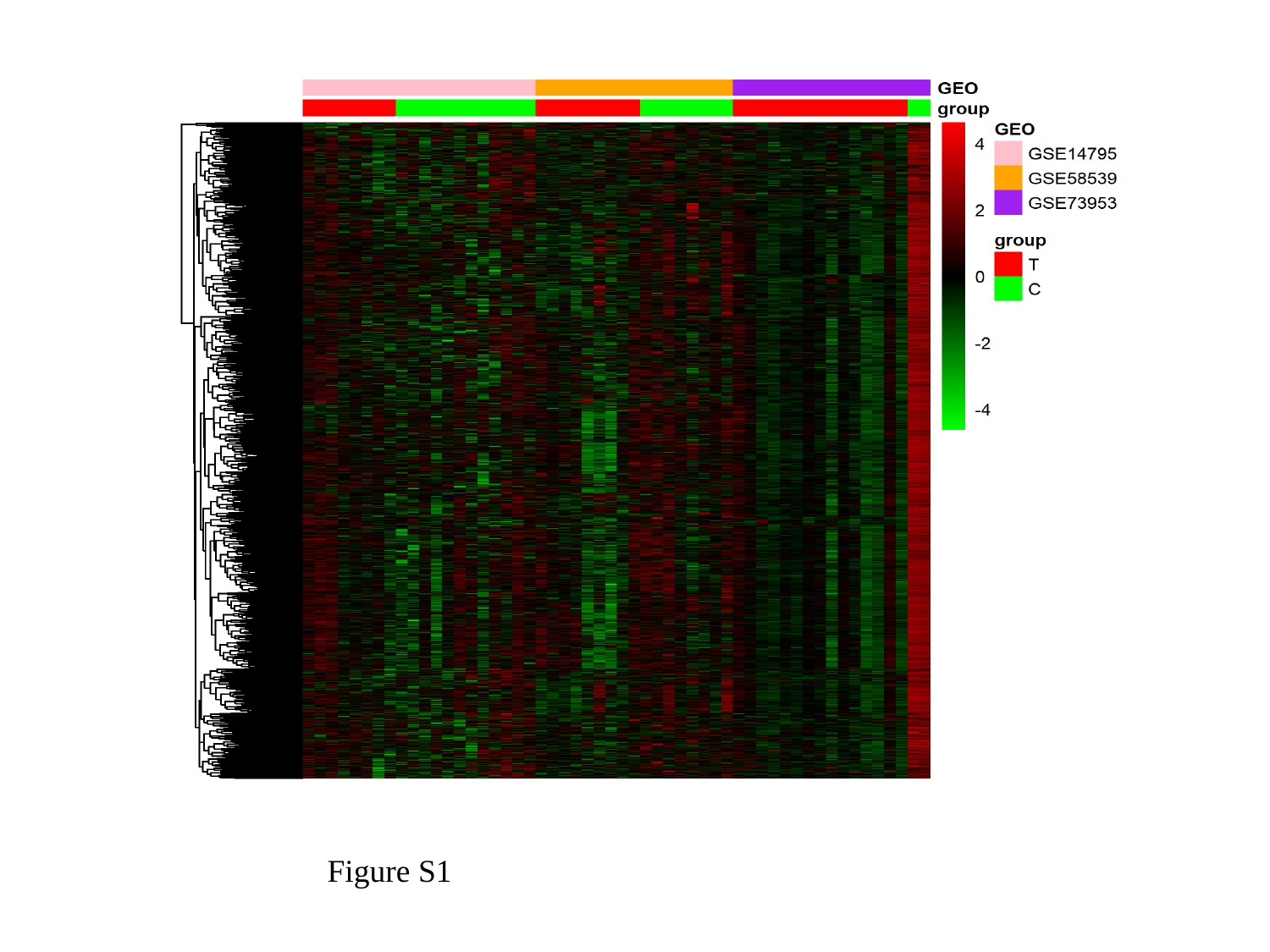

Figure S1

Supplement: Supplementary file 2 — Additional file 2: Figure S1. Heatmap illustration of the patterns of change in a particular gene across different datasets. Expression levels are represented by red (high) and green (low expression). Samples are from 35 patients with IgAN (red) and 19 healthy subjects (green). [file 12967_2019_2064_MOESM2_ESM.pptx]
